# Transcriptome Sequence of Antibiotic-Treated Pseudomonas aeruginosa

**DOI:** 10.1128/MRA.01367-18

**Published:** 2019-03-21

**Authors:** Saika Esani, Tsute Chen, Kai P. Leung, Tricia A. Van Laar

**Affiliations:** a Department of Biology, College of Science and Mathematics, California State University—Fresno, Fresno, California, USA; b The Forsyth Institute, Cambridge, Massachusetts, USA; c Dental and Craniofacial Trauma Research and Tissue Regeneration Directorate, U.S. Army Institute of Surgical Research, JBSA Fort Sam, Houston, Texas, USA; Queens College

## Abstract

Pseudomonas aeruginosa is known to tolerate antibiotic therapy during infection. This prevents clearance of infection and negatively impacts patient outcomes. Here, we report the transcriptome sequence of antibiotic-treated and untreated P. aeruginosa cultures and the differential gene expression observed when treated cells are compared to untreated cells.

## Abstract (precis)

Pseudomonas aeruginosa is known to tolerate antibiotic therapy during infection. This prevents clearance of infection and negatively impacts patient outcomes.

## ANNOUNCEMENT

Pseudomonas aeruginosa is a Gram-negative bacterium which causes various human infections using a suite of virulence factors (1). The most severe manifestations of P. aeruginosa are wound infections in burn victims and chronic lung infections in cystic fibrosis patients. P. aeruginosa is strongly associated with nosocomial infections and is known to contaminate hospital floors, beds, and medical devices (1). P. aeruginosa is a major public health concern because of its intrinsic antibiotic resistance profile and ability to tolerate antibiotic therapy (2). The ability to tolerate antibiotic therapy in the absence of resistance can result from changes in gene expression, increased lag times, and the formation of a heterogeneous population containing persister cells (2–4). Here, we report the transcriptome sequences of antibiotic-treated planktonic cultures and untreated biofilm, stationary, and planktonic cultures of P. aeruginosa to gain better insight of the potential changes in gene regulation leading to antibiotic tolerance.

An 18-h culture of P. aeruginosa PAO1-UW (5) was grown in Luria-Bertani (LB) broth at 37°C. Cells were centrifuged and washed with 0.85% NaCl three times before they were incubated with ciprofloxacin (50 µg/ml) in 0.85% NaCl (treated) or 0.85% NaCl alone (stationary) for 3.5 h (6). Following incubation, cells were pelleted and preserved in RNAprotect (Qiagen, Hilden, Germany). For the planktonic culture, the 18-hour culture was used to inoculate fresh LB broth, which was incubated until the optical density at 600 nm (OD_600_) reached 0.5, at which point the cells were collected and preserved in RNAprotect. Biofilms were formed in LB broth on borosilicate glass discs as described previously and preserved in RNAprotect (7). Each treatment condition was performed twice. RNA was isolated using the RNeasy kit according to the manufacturer’s instructions and treated twice with Turbo DNase (Ambion, Grand Island, NY) to remove any contaminating DNA (7, 8). rRNA was depleted using the Ribo-Zero magnetic kit (Epicentre, Madison, WI), and the directional library (100 bp) was generated using the NEXTflex directional RNA transcriptome sequencing (RNA-seq) kit (Bioo Scientific, Austin, TX) before sequencing using an Illumina HiSeq 2000 instrument. Following demultiplexing with CASAVA, the reads were mapped to the annotated NCBI reference sequence of strain PAO1 (no. NC_002516) using Bowtie, allowing 2 mismatches in a default seed length of 28 nucleotides to prevent the mapping of low-quality reads (9). Reads were separated into forward and reverse directions using SAMtools (10) and visualized in JBrowse (11) for verifying strand-specific transcription mapping results. Read counts per gene were calculated with the HTSeq script version 0.6.1p1 (12), and differential expression of protein-encoding genes was analyzed using the Bioconductor software package DESeq (13). Default settings were used for all software analyses. The summary of sequencing reads, mapping, and differential expression results can be found in Table 1.

**TABLE 1:** Summary of sequencing reads

Condition (replicate no.)	No. of reads (in millions)	% of reads mapped on forward strand	% of reads mapped on reverse strand	No. of upregulated ORFs^*a*^	No. of downregulated ORFs^*a*^
Treated (1)	14.6	22.5	39.3		
Treated (2)	13.8	29.0	36.0		
Stationary (1)	14.3	32.3	48.6	144	234
Stationary (2)	15.0	28.4	49.1		
Planktonic (1)	16.2	34.8	61.1	799	912
Planktonic (2)	16.0	33.7	61.6		
Biofilm (1)	16.0	36.7	57.6	67	15
Biofilm (2)	16.1	38.3	55.2		

a Differential expression in treated cells compared to other conditions.

As some of these open reading frames (ORFs) may be involved in antibiotic tolerance, these data will provide a starting point for investigating the mechanisms of tolerance in P. aeruginosa. This knowledge may assist studies of posttreatment relapse and latent infections.

### Data availability.

The RNA-seq reads and the DESeq results have been deposited in the NCBI Gene Expression Omnibus (GEO) (14) and are accessible through GEO series accession no. GSE120602.
